# Role-playing interventions in adult psychotherapy: a systematic review of clinical applications, reported outcomes, and future directions

**DOI:** 10.3389/fpsyg.2026.1749378

**Published:** 2026-04-21

**Authors:** Matteo Mazzucato, Micol Savastano, Antonio Iudici

**Affiliations:** 1 Institute of Psychology and Psychotherapy (Scuola Interazionista), Padua, Italy; 2 Department of Philosophy, Sociology, Education and Applied Psychology, University of Padua, Padua, Italy

**Keywords:** adult populations, clinical psychology, psychodrama, psychotherapy, role playing, systematic review, therapeutic outcomes

## Abstract

**Background:**

Role-playing has been employed across diverse psychotherapeutic traditions, yet no comprehensive synthesis has examined contemporary applications with adult populations and analyzed which theoretical orientations have reported beneficial or positive outcomes and under what implementation modalities.

**Objective:**

This systematic review with narrative synthesis aimed to summarize empirical evidence (2015–2025) on the use of role-playing in adult psychotherapy, examining implementation patterns (clinical populations, design and duration, descriptions of role-playing procedures), therapeutic outcomes, and emerging trends across theoretical frameworks.

**Methods:**

Following PRISMA 2020 guidelines, a systematic search was conducted in PubMed, PsycINFO, Scopus, and Web of Science for empirical studies published between January 2015 and May 2025. Eligible studies included adults (≥18 years) receiving psychotherapeutic interventions that incorporated role-playing or related enactment techniques (e.g., psychodrama, empty-chair technique, behavioral rehearsal), defined as therapeutic interventions in which the therapist assigns and guides the enactment of roles to foster psychological change.

**Results:**

Twenty-six studies met the inclusion criteria. Most (≈77–80%) adopted psychodramatic frameworks, primarily in group settings (73%), addressing conditions such as depression, trauma, substance use, chronic illness, dementia, fibromyalgia, infertility, and complicated grief. Across studies, consistent improvements were observed in symptom reduction, emotional regulation, interpersonal functioning, and empowerment, with preliminary evidence for psychophysiological changes (e.g., cortisol reduction, neural connectivity). Role-playing was most frequently conceptualized within psychodramatic or experiential models, but promising integrative applications combined psychodrama with cognitive-behavioral, emotion-focused, and person-centered approaches.

**Conclusion:**

Findings from this review suggest promising but preliminary evidence regarding the use of role-playing as a flexible and theoretically versatile intervention that can be adapted across clinical populations and orientations. Considering its reported beneficial effects across diverse clinical contexts, there is substantial potential for other therapeutic approaches to systematically integrate and investigate role-playing within their own theoretical frameworks. Future studies should therefore explore how role-playing can be used in different models of psychotherapy, employ rigorous designs, standardized outcome measures, and longitudinal assessments, and examine both in-session and out-of-session enactments to clarify processes of change and long-term effects.

## 1 Introduction

### 1.1 State of the art and research questions

Role-playing has long been employed in psychotherapy as a therapeutic intervention that enables patients to explore alternative identities, rehearse new behaviors, and consolidate psychological change through the enactment of assigned roles. Its versatility allows therapists to tailor interventions to individual client needs, making it a clinically useful and adaptable technique across therapeutic contexts. Role-playing can be implemented both in individual and group psychotherapy, and through different modalities depending on the clinician’s theoretical orientation and treatment goals.

As Corsini (1966) argued, role-playing becomes therapeutic through a dynamic process of action and reflection. It allows individuals to enhance their self-awareness regarding the effects of their ways of relating to themselves, others, and their environment, while simultaneously offering the opportunity to experiment and reinforce change. The therapeutic value of this stratagem does not lie solely in the insight derived from enacting a new behavior, but in its capacity to place the individual in a situation that elicits spontaneity and creativity (Maya et al., 2025), thereby fostering a sense of agency in relation to their problematic experience. Contemporary dialogical perspectives further conceptualize psychodramatic spontaneity not as impulsivity but as flexible readiness that enables perspective shifting and the reorganization of dominant self-positions through unscripted enactment (Mohammed et al., 2024).

Although the introduction of role-playing into psychotherapy is generally attributed to Moreno (1946) within the context of psychodrama, it has subsequently been adopted and reformulated as a mechanism of change across diverse theoretical and methodological traditions (Nardone and Salvini, 2019). The empty-chair dialogue is a method that allows patients to explore and resolve internal conflicts through an imaginary dialogue (Perls et al., 1951; Safran and Greenberg, 1987). “As-if” enactments are tools that facilitate cognitive and emotional restructuring. These techniques encourage patients to imagine and experience alternative scenarios, promoting new perspectives and solutions (Yapko, 2012). Recent developments in online psychodrama have introduced the construct of the “virtual psychodramatic role” and the notion of “hypersupplementary reality,” suggesting that digital environments may amplify rather than diminish the dramatic “as if” context (Nery, 2021). Family role enactments are used to explore and modify interactional patterns within the family system. This practice helps identify and transform dysfunctional dynamics (Selvini Palazzoli et al., 1978; Nichols and Davis, 2020). Role-playing serves as a behavioral rehearsal for social and coping skills. This technique allows patients to practice in simulated situations, improving their ability to manage real-life challenges (Lazarus, 1966; Beck, 2011). The assignment and enactment of a role can also foster the development of new identity trajectories. Within postmodern perspectives, role-playing encourages individuals to explore new ways of being and relating, promoting personal change through the expansion of the individual’s role repertoire and the capacity for flexible movement between roles (Salvini, 1998; Costa et al., 2014; Keisari, 2021; Iudici and Neri, 2024; Iudici and Della Rocca, 2025).

All these techniques, although diverse, share the goal of facilitating change through direct experience and guided reflection, offering patients practical tools to address psychological and relational challenges.

Building on these conceptual foundations, a growing body of empirical research has examined the clinical relevance and mechanisms of role-playing across psychotherapeutic orientations. Empirical findings increasingly report associations between role-playing interventions and enhanced therapeutic engagement, emotional processing, and behavioral change across settings. Early investigations demonstrated that enacted experiences can produce deeper attitude and behavior change than verbal discussion alone (Janis and King, 1954; Lazarus, 1966). More recent work has shown that structured role enactments enhance interpersonal functioning and promote self-insight across diverse clinical populations, including medical and palliative contexts (e.g., Menichetti et al., 2016; Keisari and Palgi, 2016). Meta-analytic evidence indicates that role induction procedures, in which clients actively enact anticipated therapeutic experiences, significantly reduce premature termination and improve both in-session engagement and posttreatment outcomes (Swift et al., 2023). Experimental studies further demonstrate that role reversal, allowing clients to alternate between self and other perspectives, produces greater reductions in social anxiety and increases in empathy than standard role-playing conditions (Abeditehrani et al., 2020). Systematic evidence supports the efficacy of psychodrama–an established role- playing–based modality–across adult populations, demonstrating improvements in psychological well-being, interpersonal functioning, and emotion regulation (Orkibi and Feniger-Schaal, 2019). Recent integrative reviews have further documented the expanding clinical applications of psychodrama and drama-based interventions across trauma-related conditions, anxiety disorders, and medical contexts (Ciołek et al., 2022). These findings are echoed in studies highlighting role-playing’s effectiveness in fostering communication and empathy skills among trainees in mental health and medical education (Rønning and Bjørkly, 2019), as well as in tele-based adaptations developed during the COVID- 19 pandemic (Biancalani et al., 2021; Biolcati et al., 2023).

Taken together, these findings demonstrate the versatility and adaptability of role-playing as a psychotherapeutic process for which growing empirical support has been reported. Despite substantial empirical support, there remains a lack of an up-to-date systematic synthesis examining the use of role-playing in psychotherapy with adult populations.

Furthermore, no review currently provides an integrated analysis of how role-playing has been implemented across therapeutic contexts–specifically, regarding the clinical populations involved, the design and duration of interventions, the theoretical frameworks adopted, the characteristics of role-playing procedures found effective, and the primary therapeutic outcomes reported.

The review was guided by the following overarching question: how are role-playing interventions employed in adult psychotherapy, and what therapeutic outcomes and implementation patterns are reported in the empirical literature from 2015 to 2025.

A secondary question examined how different theoretical frameworks have conceptualized and applied role-playing interventions associated with reported therapeutic outcomes.

Considering these gaps, this systematic review aims to:

1. Systematically map and synthesize reported therapeutic outcomes of role-playing across different theoretical orientations;
2. Examine patterns of implementation, including clinical populations, intervention design and duration, and descriptions of role-playing procedures;
3. Identify the theoretical frameworks that have conceptualized and employed role-playing as a therapeutic technique;
4. Highlight emerging trends, research gaps, and future directions relevant to contemporary clinical practice.

By systematically mapping contemporary evidence, this review aims to contribute to the advancement of integrative, evidence-based practices in clinical psychology.

### 1.2 Theoretical background

From a conceptual standpoint, the present review adopts an interactionist framework (Salvini, 1998; Salvini and Dondoni, 2011; Iudici et al., 2020), according to which a “role” is understood as a relational positioning process through which individuals assume a specific location in relation to an interlocutor. Such positioning emerges from dynamic processes of self- and hetero-attribution, as well as from normative prescriptions that constrain ways of being and acting within a given relational context. For the sake of terminological coherence, the hyphenated form “role-playing” is adopted throughout the manuscript. This choice reflects its conceptualization as a theoretically grounded and unitary enactment-based clinical construct, rather than a generic combination of “role” and “playing.”

Within this perspective, role-playing is not conceived as an atheoretical technique, but as a clinical device whose therapeutic potential depends on the theoretical orientation of the practitioner employing it. The transformative value of role assumption does not reside in the mere execution of a structured procedure, but in the possibility of exploring, destabilizing, or consolidating patterns of relational positioning through enacted experience.

At the same time, in continuity with classical psychodramatic theory and subsequent experiential traditions (Moreno, 1946; Corsini, 1966), role-playing was operationally defined as involving at least one of the following core processes:

- explicit role assumption or role reversal;
- enactment of personally meaningful interpersonal scenarios;
- dialogical interaction between parts of the self or between self and imagined others;
- emotional engagement through embodied action rather than exclusively verbal narration.

Importantly, for the purposes of the present review, role-playing was considered central to an intervention when it constituted a core therapeutic mechanism contributing directly to emotional, cognitive, or interpersonal change, rather than a warm-up strategy or illustrative technique. Interventions in which role-playing was used solely for psychoeducational, training, or recreational purposes, without explicit therapeutic intent or emotional processing, were not considered eligible. This conceptual and operational framework guided both the eligibility criteria and the classification of included studies. Although role-playing is embedded in multiple theoretical traditions, the present review focuses on shared enactment-based processes within a structured therapeutic context, thereby identifying common mechanisms across orientations without presupposing theoretical homogeneity.

## 2 Methods

### 2.1 Study design

This systematic review was conducted in accordance with the PRISMA 2020 guidelines (Page et al., 2021). A systematic review design with narrative synthesis was chosen to ensure comprehensive and transparent identification, appraisal, and synthesis of empirical studies. This approach allowed the examination of heterogeneous interventions, populations, and outcomes without restricting the analysis to quantitative effect size estimation. A systematic search was conducted across PubMed, Scopus, PsycINFO, and Web of Science for peer-reviewed studies published between January 2015 and May 2025 in English. This time frame was selected to capture contemporary applications of role-playing in psychotherapy. Search terms combined four concept clusters–role-playing interventions, psychotherapy, adult populations, and clinical outcomes–using Boolean operators.

### 2.2 Eligibility criteria

For the purposes of this systematic review, role-playing intervention is defined as a psychotherapeutic procedure in which patients are explicitly assigned and guided by a therapist to enact a role, either their own or that of a significant other, within a structured therapeutic framework, with the explicit aim of producing psychological change (e.g., emotional processing, behavioral modification, identity reorganization, interpersonal change). Consistent with the introduction and the theoretical premises, the present review included psychotherapeutic interventions in which role-playing was explicitly employed as part of the therapeutic process with adult participants. Specifically, our operationalization encompassed a set of labels frequently used in the literature to refer to enactment-based therapeutic procedures, including: role enactment, identity enactment, acting as-if, assumption of a prescriptive role, strategic enactment, role reversal, dramatization, empty chair, behavioral rehearsal, psychodramatic intervention, and drama-based intervention. Search terms were developed through a preliminary scoping search and refined by cross-checking terminology used across theoretical traditions (e.g., psychotherapy dictionaries/handbooks and prior empirical literature). Although these approaches are variably labeled across theoretical traditions, they share the same underlying therapeutic structure: the assignment, assumption, enactment, and/or experiential exploration of roles within a clinical frame and with explicit therapeutic intent. Differences across these devices primarily concern the setting (individual vs. group; in-person vs. remote), the degree of structure, and the theoretical orientation. However, the core transformative process remains common: enacting and experimenting with alternative roles to facilitate cognitive, behavioral, and emotional development. Role-playing was considered present only when enactment constituted a core and recurring component of the intervention (i.e., explicitly described as a primary therapeutic strategy or recurring procedural element), rather than a brief illustrative, educational, or adjunct exercise (e.g., as a brief demonstration, warm-up exercise, or peripheral technique). Interventions were not considered role-play and were therefore excluded when they consisted solely of: (a) imagery-based or visualization exercises without enacted performance; (b) generic psychoeducational role-play vignettes used only once or as illustrative examples; or (c) brief communication or skills-rehearsal exercises lacking therapeutic processing, emotional exploration, or integration within an ongoing psychotherapeutic framework. Notably, eligibility criteria were intentionally restricted to interventions delivered within a psychotherapeutic framework and targeting clinical or health-related psychological difficulties, in order to enhance conceptual specificity and clinical relevance.

#### 2.2.1 Inclusion criteria

Studies were included if: (a) Clinical population: adults (≥18 years) receiving psychotherapy or psychological treatment for clinical or health-related psychological difficulties; (b) Setting: individual or group psychotherapy; in-person or remote; (c) Intervention type: enactment-based psychotherapeutic interventions involving therapist-guided role assumption and enactment; (d) Role of role-playing: role-playing/enactment must represent a central procedural component of the intervention; (e) Type of study: reported empirical data (quantitative, qualitative, or mixed-methods); and were peer-reviewed and published in English between 2015 and 2025; (f) Outcomes: any clinical/psychological outcomes reported.

#### 2.2.2 Exclusion criteria

Studies focusing solely on educational simulations, professional skills training, or recreational role-playing games were excluded. Studies were excluded if they: (a) involved only minors or non-clinical training simulations; (b) were purely theoretical or opinion papers; (c) consisted of conference abstracts, dissertations, or gray literature. Studies examining role-playing games, avatar-based simulations, or online recreational role-play–such as role-playing Games (RPGs) and Massively Multiplayer Online role-playing Games (MMORPGs)–were excluded because these activities do not meet the operational definition of therapeutic role-playing adopted in this review, which requires the assignment and enactment of roles within a structured psychotherapeutic intervention. In summary, interventions were excluded when role-playing was limited to: (a) educational or professional training simulations without a clinical aim; (b) recreational or game-based role-playing activities (e.g., RPGs, MMORPGs); (c) isolated illustrative exercises without therapeutic processing; (d) non-therapist-guided activities lacking a defined psychotherapeutic framework.

#### 2.2.3 Search strategy

We adopted a database-driven, predefined-search approach to ensure procedural reproducibility and minimize *post hoc* expansion of the corpus. We acknowledge that the absence of citation chasing or hand-searching may have reduced sensitivity. To enhance coverage despite not performing citation chasing, we used four complementary databases indexing clinical, psychological, and interdisciplinary mental health research (PubMed, PsycINFO, Scopus, and Web of Science) and employed an intentionally broad cluster of enactment-related terms (e.g., psychodrama, empty-chair, behavioral rehearsal, role reversal). In addition, the final set of included studies was cross-checked to ensure that their keywords and indexing terms were represented in the search strategy. Although citation chasing was not performed, sensitivity was maximized through (a) four complementary databases, (b) broad enactment-related terms, and (c) cross-checking indexing terms of included papers. Nevertheless, some relevant studies may have been missed. Database searches were conducted between 14 October 2025 and 17 October 2025: PubMed (14 October 2025), Scopus (16 October 2025), and Web of Science and PsycINFO (17 October 2025). The filters were applied only to the Scopus and PsycINFO databases. The following filters were applied in PsycINFO: language: English, age group: adulthood, young adult, thirties, middle aged, aged, very old, years of publication (2015–2025). Filters used on Scopus were: language: English, years of publication (2015–2025).

Search strings used:

Pubmed

(“role play”[tiab] OR “role playing”[tiab] OR “role - playing”[tiab] OR “role enactment”[tiab] OR “identity enactment”[tiab] OR “acting as if”[tiab] OR “as-if technique”[tiab] OR “prescriptive role”[tiab] OR “strategic enactment”[tiab] OR “role reversal”[tiab] OR dramatization[tiab] OR “role playing technique”[tiab] OR “empty chair”[tiab] OR “behavioral rehearsal”[tiab] OR psychodrama[tiab] OR psychodramatic[tiab] OR “drama therapy”[tiab] OR “drama-based”[tiab]) AND (psychotherapy[tiab] OR psychotherapeutic[tiab] OR “psychological treatment”[tiab] OR “psychological intervention”[tiab] OR “clinical psychology”[tiab] OR “counseling psychology”[tiab] OR psychology[tiab] OR psychological[tiab] OR “psychological technique”[tiab]) AND (adult*[tiab] OR patient*[tiab] OR client*[tiab] OR “clinical population”[tiab]) AND (“treatment outcome*”[tiab] OR outcome*[tiab] OR effect*[tiab] OR efficac*[tiab]OR “therapeutic change”[tiab] OR “symptom reduction”[tiab] OR symptomatology[tiab] OR “clinical outcome”[tiab] OR “psychotherapeutic outcome”[tiab] OR “quality of life”[tiab] OR wellbeing[tiab] OR change[tiab]) AND (“2015”[dp]: “2025”[dp]) AND english[lang].

Web of Science

TS = (“role - play” OR “role playing” OR “role - playing” OR “role enactment” OR “identity enactment” OR “acting as if” OR “as-if technique” OR “prescriptive role” OR “strategic enactment” OR “role reversal” OR dramatization OR “role playing technique” OR “empty chair” OR “behavioral rehearsal” OR psychodrama OR psychodramatic OR “drama therapy” OR “drama-based”) AND TS = (psychotherapy OR psychotherapeutic OR “psychological treatment” OR “psychological intervention” OR “clinical psychology” OR “counseling psychology” OR psychology OR psychological OR “psychological technique” OR “psychological intervention”) AND TS = (adult* OR patient* OR client* OR “clinical population”)

AND TS = (“treatment outcome*” OR outcome* OR effect* OR efficac* OR “therapeutic change” OR “symptom reduction” OR symptomatology OR “clinical outcome” OR “psychotherapeutic outcome” OR “quality of life” OR wellbeing OR change) AND PY = (2015–2025) AND LA = (English)

Psychinfo

XB (XB (“role - play” OR “role playing” OR “role - playing” OR “role enactment” OR “role reversal” OR dramatization OR “role playing technique” OR “empty chair” OR “behavioral rehearsal” OR psychodrama OR “drama therapy” OR “drama-based”) AND (psychotherapy OR psychotherapeutic OR “psychological treatment”) AND (adult* OR patient* OR client* OR “clinical population”) AND (“treatment outcome*” OR outcome* OR effect* OR efficac*OR “therapeutic change” OR “symptom reduction” OR symptomatology OR “clinical outcome” OR “psychotherapeutic outcome” OR “quality of life” OR wellbeing OR change)

Scopus

TITLE-ABS-KEY (“role - play” OR “role playing” OR “role - playing” OR “role enactment” OR “role reversal” OR dramatization OR “role playing technique” OR “empty chair” OR “behavioral rehearsal” OR psychodrama OR”drama therapy” OR “drama-based”) AND TITLE-ABS-KEY (psychotherapy OR psychotherapeutic OR”psychological treatment”) AND TITLE-ABS-KEY (adult* OR patient* OR client* OR “clinical population”) AND TITLE-ABS-KEY (“treatment outcome*” OR outcome* OR effect* OR efficac*or “therapeutic change” OR”symptom reduction” OR symptomatology OR “clinical outcome” OR “psychotherapeutic outcome” OR “quality of life” OR wellbeing OR change) AND (EXCLUDE (EXACTKEYWORD, “adolescent”) OR EXCLUDE (EXACT KEYWORD, “child”) OR EXCLUDE (EXACT KEYWORD, “training”) OR EXCLUDE (EXACT KEYWORD, “learning”) OR EXCLUDE (EXACT KEYWORD, “school child”)

### 2.3 Study selection and data extraction

**Figure 1:**
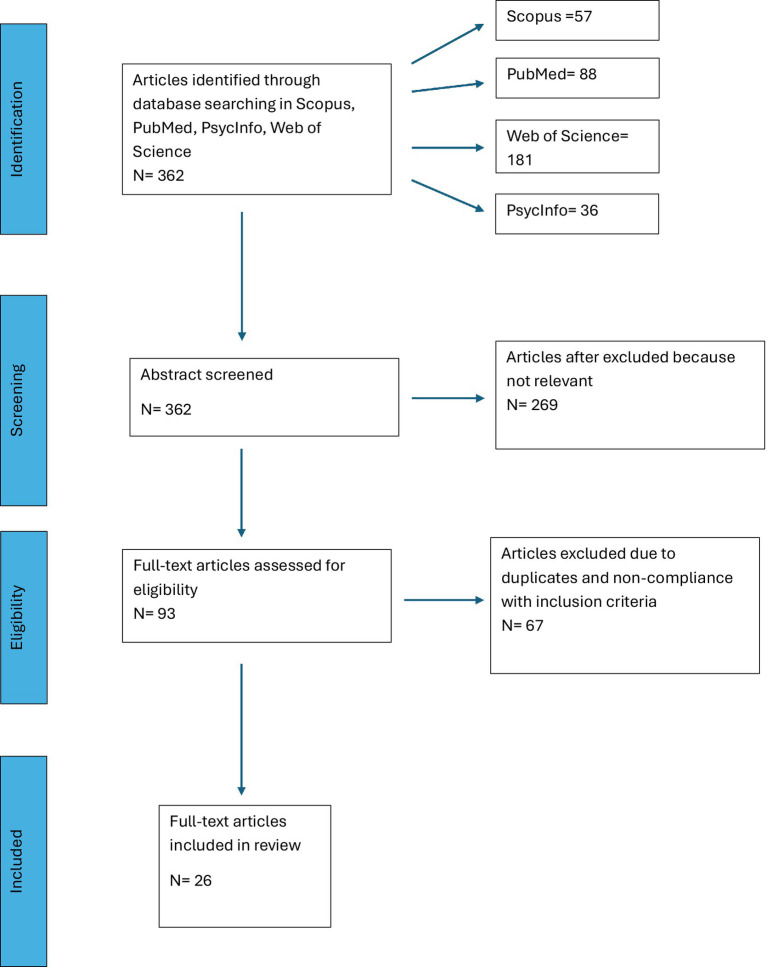
PRISMA flow diagram illustrating the processes of systematic literature searches and screening.

All retrieved records were compiled and managed in a spreadsheet using Google Sheets (Google LLC; web-based version, accessed 2 October 2025). Duplicate records were identified and removed prior to screening through manual checking of titles, authors, publication year, and source information. Titles and abstracts were independently screened by two reviewers according to the eligibility criteria, with screening decisions recorded in dedicated spreadsheet columns. Full-text eligibility assessment was performed independently by the same reviewers and documented in the same file. Disagreements were resolved through discussion and, when necessary, by consultation with a third reviewer. A standardized data extraction form (spreadsheet-based) was developed *a priori* to systematically collect key information from each included study. Extracted data included: study identification (title, authors, publication year); clinical population (as reported in the study); intervention characteristics (format—individual or group; setting; duration; frequency); theoretical orientation (explicit therapeutic model or framework); description of the role-playing/enactment procedures; and main findings and reported outcomes (e.g., symptom- related, behavioral, emotional, or interpersonal changes). Data extraction was conducted independently by two reviewers and verified by a third to ensure completeness and accuracy. The manuscript was drafted in Microsoft Word (Microsoft Corporation). The PRISMA 2020 flow diagram was created manually based on the documented screening counts (i.e., no dedicated PRISMA diagram software was used). To enhance conceptual clarity, the role of role-playing within each intervention was classified as either core or adjunct. Role-play was coded as a core component when it was explicitly described by the authors as the primary therapeutic mechanism, technique, or change process of the intervention, or when it constituted a recurring and central feature across treatment sessions. In contrast, role-play was classified as an adjunct technique when it was used only briefly or occasionally, was not described as integral to the therapeutic model, or was not directly linked to the main therapeutic processing or change mechanisms of the intervention. This distinction was applied consistently during data extraction based on explicit descriptions provided in the original studies. In addition, specific variables were extracted to characterize the enactment-based nature of the interventions. These included the centrality of role-playing within the intervention (coded as core or adjunct), the specific enactment techniques employed (e.g., role reversal, empty-chair work, dramatization), and the format and dose of enactment procedures. This coding was based exclusively on explicit descriptions provided by the study authors and was applied consistently across studies (see Figure 1).

### 2.4 Data synthesis

Due to the methodological diversity across study designs and outcome measures, a narrative and thematic synthesis was conducted. As a consequence, the review does not attempt to derive pooled effect size estimates. Accordingly, the present review should be understood as a systematic mapping and critical narrative synthesis of reported outcome patterns rather than as a formal effectiveness evaluation. The synthesis process aimed to: (1) Map the range and characteristics of role-playing interventions in adult psychotherapy; (2) Identify theoretical and procedural variations across studies; (3) Summarize reported clinical outcomes and therapeutic impacts; and (4) Highlight consistencies, gaps, and emerging trends across theoretical orientations. Detailed characteristics of all included studies are provided in Table 1. Risk of bias was formally assessed using design-appropriate tools (see Section 2.5 and Table 2) and was considered in interpreting findings. This review was prospectively registered in PROSPERO (ID 1334147). The review framework, including eligibility criteria, operational definitions, databases, time frame, and outcome domains, was established prior to study screening and documented before the selection process began. These criteria were applied consistently throughout the review, and no modifications to eligibility criteria or search strategy were introduced after screening commenced.

**Table 1:** Characteristics of included studies.

No.	References	Country	Clinical population	Study design and intervention duration	Theoretical framework/intervention	Centrality (core/integrated/adjunct)	Role-play methods (coded labels)	Format	Dose	Main findings / outcomes
1	Menichetti et al. (2016)	Italy	Adults with cancer; qualitative interview sample *n* = 8 (mean age 59.25 years; range 47–73), drawn from two ongoing psychodrama groups (total group pool *n* = 21).	Qualitative study using Interpretative Phenomenological Analysis (IPA); semi-structured interviews; no control group.	Psychodrama (Moreno-based group psychotherapy)	Core (psychodrama group participation was the focus of the study)	Warm-up, action, and sharing phases; role reversal explicitly described; mirroring reported as a related experiential effect.	Group; In-person	Warm-up, action, and sharing phases; role reversal explicitly described; mirroring reported as a related experiential effect.	Qualitative themes indicated that group psychodrama supported cancer adjustment, including enhanced emotional expression and processing, changes in identity and self-perception, and increased meaning-making; no quantitative outcomes reported.
2	Biolcati et al. (2017)	Italy	University students with self-reported mental health problems; *N* = 30 (22 women, 8 men; mean age 22.3 years).	Uncontrolled pre–post study; no control group.	Analytical psychodrama, integrating Moreno’s psychodramatic method with psychoanalytic and group-dynamic principles (Kleinian and Bionian traditions).	Core	Role enactment; role reversal; classical psychodramatic action within an analytically oriented group framework.	Group, in-person	40 weekly group sessions; 90 min each; total duration approximately 1 year.	Preliminary pre–post improvements in psychological functioning were reported, suggesting that analytical psychodrama may function as a supportive psychotherapeutic intervention for university young adults; no controlled comparisons reported.
3	Biolcati et al. (2023)	Italy	Young adults with anxiety–depressive psychological difficulties; (*N* = 22 participants across three online groups).	Uncontrolled pre–post (test–retest) observational study; no control group.	Analytic psychodrama (group psychotherapy delivered via videoconferencing).	Core (analytic psychodrama constituted the intervention model).	Role reversal; doubling; solo; observation with feedback; enactment-based analytic group work explicitly described as recurring session components.	Group; Tele-based	Weekly group sessions, 90 min each; approximately 10 months of treatment; 38 sessions in total; group size 7–8 participant.	Significant pre–post improvements on general psychological distress (CORE-OM) were reported over the 10-month intervention period; changes in alexithymia were also reported, although detailed statistics were not consistently provided
4	Elkarif et al. (2025)	Israel	Community-dwelling older adults with constricted life-space mobility; age range 63–102 years; *N* = 111 completers.	Randomized controlled trial (parallel-group design); tele-based intervention with control condition.	Drama therapy (tele-drama therapy)	Core (enactment-based drama therapy constituted the primary therapeutic intervention).	Playback theatre (story enactment); dramatic enactment; group-based narrative dramatization	Group, tele-based (online)	Weekly group sessions, 75 min each, over 12 weeks	Significant improvements were reported in social connectedness, personal growth, and overall well-being, alongside reductions in depressive symptoms in the intervention group
5	Şener (2022)	Turkey	Women diagnosed with fibromyalgia; *N* = 9; adult clinical sample	Uncontrolled pre–post study; no control group	Classical psychodrama (Moreno-based group psychotherapy)	Core (psychodrama constituted the primary therapeutic intervention)	Doubling; mirroring; dramatization; warm-up; sharing phase	Group, in-person	Weekly group sessions; 12 sessions total; 120 min each	Reductions in pain levels, improved quality of life, and increased ability to express emotions were reported following the psychodrama intervention
6	Gamoneda et al. (2025)	NR	IIndividuals with complicated grief; *N* = 26; adult clinical sample receiving telepsychotherapy.	Open trial with uncontrolled pre–post and follow-up assessment; individual teletherapy format; no control group	Emotion-Focused Therapy (EFT) with chair work	Core (the empty-chair task constituted the primary in-session experiential technique and the focus of the process–outcome analysis).	Empty-chair task for unfinished business; EFT chair work involving role enactment and dialogical processing	Individual, tele-based (online).	Three sessions in total; empty-chair task implemented in session 2; follow-up assessment at 2 months.	Large pre–post and follow-up reductions in grief symptomatology were reported; clinically significant change observed in 36% of participants at follow-up. Resolution processes within the empty-chair task predicted symptom improvement at post-treatment and follow-up
7	Testoni et al. (2020)	Italy	Adults with substance dependence residing in an attenuated custody institution in Northern Italy; *N* = 7	Mixed-methods study using an idiographic change process approach; uncontrolled pre–post quantitative assessment combined with qualitative process analysis; no control group.	Psychodrama (Moreno-based group psychotherapy) delivered within an institutional treatment program	Core (psychodrama constituted the primary therapeutic intervention).	Mirroring; doubling; role reversal; soliloquy; auxiliary ego work; protagonist-centered enactments.	Group; in-person	21 weekly psychodrama sessions; session duration ranged from 90 min to 2 h	Pre–post improvements were reported on measures of psychological distress (CORE-OM), spontaneity (SAI-R), perceived self-efficacy (GSE), and alexithymia (TAS-20). Qualitative analyses (Change Coding System; Helpful Aspects of Therapy) documented perceived therapeutic change processes.
8	Biancalani et al. (2021)	Italy	Adults participating in two ongoing psychodrama groups; qualitative interview sample *n* = 15 (mean age 40.73 years, SD = 11.07).	Qualitative study focusing on clients’ helpful and hindering experiences; no control group.	Psychodrama (Moreno-based group psychotherapy) adapted to an online format during the COVID-19 pandemic.	Core (tele-psychodrama sessions constituted the focus of the study).	Role reversal; doubling; soliloquy; psychodramatic enactments adapted for online delivery.	Group, tele-based (online).	Weekly group sessions transitioned to an online format from March to May 2020; 12 tele-psychodrama sessions.	Qualitative themes described perceived helpful and hindering aspects of tele-psychodrama, including experiences of emotional expression, group connection, and perceived limitations of the online format; no quantitative outcomes reported.
9	Testoni et al. (2018)	Italy	Inpatients in a therapeutic community for substance addiction in Northern Italy; initial group *N* = 9 (female participants, mothers; age range 24–50 years); idiographic case series focused on 4 participants who completed the program.	Mixed-methods idiographic case series using Change Process Research (CPR); uncontrolled pre–post quantitative assessment combined with qualitative process analysis; no control group.	Psychodrama integrated with the Person-Centered Approach (PCA) within a therapeutic community setting	Core (psychodrama constituted the primary therapeutic intervention)	Warm-up; protagonist-centered dramatizations; mirroring; doubling; soliloquy; role reversal; storytelling; social atom; final sharing	Group; In-person	Weekly group sessions delivered over 6 months; each session lasted approximately 2 h.	Quantitative pre–post improvements were reported on measures of psychopathology (MMPI), psychological distress (CORE-OM), spontaneity (SAI-R), perceived self-efficacy (GSE), and well-being (Pro. Spera). Qualitative analyses (Helpful Aspects of Therapy; Client Change Interview) identified change processes related to increased self-awareness, empowerment, and relational functioning.
10	Bardideh et al. (2025)	Iran	Bereaved adults aged 18–50 years diagnosed with Prolonged Grief Disorder following COVID-19–related bereavement (≥6 months since loss); *N* = 36 (intervention *n* = 18; control *n* = 18).	Randomized controlled trial (parallel-group design) comparing an active intervention with a no-treatment control; post-treatment and 2-month follow-up assessments.	Integrated group therapy combining cognitive-behavioral group therapy and Gestalt therapy (empty-chair technique), based on the Kim Paleg grief protocol.	Integrated (role-playing enactment via empty-chair work embedded within a broader CBT-based group therapy protocol).	Empty-chair technique (Gestalt); imaginal dialogue with the deceased; expressive enactments of unresolved emotions (e.g., guilt, anger); letter-writing followed by enactment; farewell/saying-goodbye enactments.	Group, in-person	16 group sessions, 90 min each, delivered twice weekly over 8 weeks.	Compared with the control group, the intervention group showed significant reductions in depression (BDI-II), anger (NAI), and guilt/shame (GASP) at post-treatment (*p* < 0.001), with effects maintained at 2-month follow-up. Prolonged grief was not directly assessed as a construct; outcomes reflected associated emotional symptoms.
11	Erbay et al. (2018)	Turkey	Adults aged 18–65 years diagnosed with major depressive disorder (DSM-5); *N* = 10 enrolled, *N* = 8 completers.	Single-group uncontrolled pre–post study; no control group.	Psychodrama (Moreno-based group psychotherapy) delivered within a university hospital psychiatry clinic.	Core (psychodrama constituted the primary therapeutic intervention).	Group psychodramatic enactments conducted by a therapist and co-therapist under supervision; specific psychodramatic techniques not itemized (NR).	Group; In-person	Weekly group sessions over 16 weeks; session duration approximately 3 h	Significant pre–post reductions were reported in perceived stress (PSS), anxiety (STAI-1/2), depressive symptoms (BDI), and salivary cortisol levels following the psychodrama intervention.
12	Giacomucci et al. (2025)	USA	Inpatients in residential addiction treatment with co-occurring disorders; *N* = 148.	Mixed-methods study conducted within an inpatient trauma track; uncontrolled pre–post quantitative assessments combined with qualitative exit-survey content analysis; no control group.	Trauma-focused psychodrama (Moreno-based action methods integrating psychodrama, sociometry, and group sharing).	Core (psychodrama constituted the primary therapeutic intervention).	Experiential sociometry; full psychodrama sequence including warm-up, sociometric topic selection, protagonist selection, enactment/scene work, and group sharing.	Group; In-person	Approximately two sessions per week (≈4.5 h/week); average attendance 4.63 sessions per participant (range 1–8)	Substantial pre–post reductions were reported in PTSD (−52.71%), depression (−61.76%), anxiety (−53.72%), traumatic grief (−31.39%), and prolonged grief disorder (−32.36%), alongside increased spontaneity (+31.08%). High levels of patient satisfaction were also reported.
13	Keisari and Palgi (2016)	Israel	Older adults aged 62–93 years; *N* = 55 (intervention group *n* = 27; care-as-usual control group *n* = 28).	Non-randomized controlled before–after study comparing an intervention group with a care-as-usual control group.	Drama therapy integrating life review and playback theatre techniques.	Core (drama-based enactment constituted the primary therapeutic intervention).	Playback enactment; autobiographical vignettes; role-taking within a life-review framework.	Group, in-person	Playback enactment; autobiographical vignettes; role-taking within a life-review framework.	Significant time × group interaction effects were reported, indicating increased meaning in life, self-acceptance, quality of relationships with others, and successful aging, alongside reduced depressive symptoms in the intervention group compared with care-as-usual.
14	Yu et al. (2022)	China	Adults with childhood trauma–associated major depressive disorder receiving first-line antidepressant treatment; *N* = 46 (psychodrama group *n* = 29; control group *n* = 17)	Randomized controlled trial comparing antidepressants combined with psychodrama versus antidepressants combined with general health education; 6-month intervention period.	Psychodrama (Yi Shu psychodrama model) delivered as a structured group psychotherapy adjunct to pharmacotherapy.	Core (psychodrama constituted the active psychotherapeutic intervention).	Classical psychodrama structure (warm-up, action/enactment, sharing/integration) with stabilization components; role enactment; role reversal; doubling; mirroring; soliloquy; protagonist-centered work.	Group, in-person; closed groups (6–10 participants).	Three intensive psychodrama intervention blocks delivered over 6 months; each block consisted of 4 consecutive days, administered once every 2 months (total of 3 blocks).	Depressive and anxiety symptoms (HAMD-17, BDI-13, BAI-21) decreased in both groups. Improvements in coping style were observed only in the psychodrama group. Neuroimaging analyses (rs-fMRI) showed significant treatment × time effects, with increased functional connectivity (right superior parietal gyrus–left inferior frontal gyrus) in the psychodrama group.
15	Terzioğlu and Özkan (2018)	Turkey	Women experiencing infertility; *N* = 30 (two closed groups of 15 participants); attrition reported.	Mixed-methods study with uncontrolled pre–post quantitative assessments; non-random group allocation; no control group.	Psychodrama (Moreno-based group psychotherapy).	Core (psychodrama constituted the primary therapeutic intervention).	Group psychodrama games conducted within the classical psychodramatic structure (warm-up, action, sharing).	Group; In-person	Eight-week intervention; approximately 3 h of group psychodrama per week	Significant pre–post improvements were reported in depressive symptoms (BDI), hopelessness, and self-esteem. Anxiety scores decreased but did not reach statistical significance.
16	Gulassa et al. (2025_)_	Brazil	Adults with DSM-5 excoriation (skin-picking) disorder receiving outpatient psychiatric care; *N* = 53 randomized (psychodrama group therapy *n* = 26; support group therapy *n* = 27); *N* = 48 completers.	Randomized controlled trial (parallel-group design) comparing psychodrama group therapy with an active control condition (support group therapy).	Psychodrama group therapy (Moreno-based enactment psychotherapy).	Core (psychodramatic enactment constituted the primary therapeutic intervention)	Role enactment; empty-chair work; concretization; internal psychodrama; role exploration; future projection	Group, in-person	Weekly group sessions; 15 sessions total; 90 min each	No significant between-group differences were observed in excoriation disorder severity. Both the psychodrama and support group therapy conditions showed improvements in psychosocial and emotional outcomes from pre- to post-treatment.
17	Doomen (2018)	Netherlands	Outpatients with Cluster C personality disorders (avoidant, dependent, obsessive-compulsive); comorbid anxiety, mood symptoms, and psychological trauma reported; *N* = 8 (5 women, 3 men; mean age 34 years, range 23–45)	Single-group exploratory study with uncontrolled pre–post assessment; no control group	Schema-focused drama therapy, integrating drama therapy and psychodrama-derived enactment techniques within a schema therapy framework	Core (drama therapy protocol constituted the tested psychotherapeutic intervention)	Drama therapy enactments within schema-focused work, including role playing, dramatized internal dialogue, imagery and rescripting, and psychodrama-derived techniques explicitly described (e.g., role reversal, mirroring, doubling)	Group, in-person	Intervention duration of approximately 3 months; number of sessions, session length, and session frequency NR.	Significant pre–post changes in schema modes were reported, including increased emotional expression and healthy modes and reduced maladaptive coping. Effects were reported as moderate to large (e.g., Cohen’s d ≈ 0.70–0.75) for pre–post comparisons, with smaller effects observed for within-treatment versus post-treatment observational indices
18	Abeditehrani et al. (2020)	Iran	Adult women with DSM-IV social anxiety disorder; *N* = 5 analyzed (mean age 36.6 years, range 21–63; generalized SAD *n* = 3, specific SAD *n* = 2).	Uncontrolled pilot trial with pre–post assessment and session-by-session monitoring; no control group.	IIntegrated cognitive behavioral group therapy and psychodrama (CBPT), combining CBT techniques with repeated psychodramatic enactments	Integrated (psychodrama techniques embedded repeatedly within a CBT group structure; sessions 3–11 followed classical psychodrama stages).	Psychodrama stages (warm-up, action, sharing); enactment of anxiety-provoking situations; role reversal; doubling; mirroring; empty-chair work; soliloquy; CBT cognitive restructuring integrated during enactments.	Group, in-person (two therapists; stage/chair setup explicitly described).	12 weekly group sessions, approximately 2.5 h each, plus an initial individual orientation interview	Significant reductions were reported in fear of negative evaluation (BFNE) and social anxiety severity (LSAS), as well as decreases in probability and cost estimates (OPQ/OCQ). No significant pre–post changes were observed in avoidance (SADS), spontaneity (PAS), depressive symptoms (BDI), or quality of life (QOLI).
19	Keisari et al. (2023)	Israel	Older adults experiencing unresolved grief related to the sudden death of a significant other (very remote loss); two evidence-based case studies drawn from a larger drama therapy group program.	Evidence-based case study approach embedded within a randomized controlled study of 12-week drama therapy groups; mixed-methods design including pre–post–follow-up self-report measures and qualitative analyses of video-recorded sessions and post-intervention interviews.	Drama therapy integrating Playback Theater with a life review paradigm; meaning reconstruction framework used to conceptualize and interpret change processes.	Core (drama-based enactment constituted the primary therapeutic mechanism).	Playback Theater–based enactments of participants’ personal stories within a life-review structure; dramatization of unfinished business across event story, back story, and personal story.	Group; in-person	Twelve-week protocol with weekly group sessions.	Case-level analyses indicated clinically significant improvements in depressive symptoms and psychological well-being at post-treatment and follow-up in one case, and improvements in self-esteem and relationship satisfaction at post-treatment (not maintained at follow-up) in the second case.
20	Giacomucci et al. (2022)	USA	Inpatients in a residential drug and alcohol treatment center enrolled in a specialty trauma track; *N* = 68 (Cohort 1 pre-pandemic *n* = 48; Cohort 2 during-pandemic *n* = 20).	Naturalistic mixed-methods pre–post study comparing two non-randomized cohorts (pre-COVID-19 vs. during COVID-19); no randomized control group; qualitative exit survey included.	Trauma-focused psychodrama group psychotherapy (Moreno-based) delivered within an inpatient trauma program alongside standard residential treatment services.	Core (psychodrama programming constituted the focal intervention evaluated).	Group psychodrama programming incorporating sociometry, psychodrama warm-ups, and a full psychodrama enactment session followed by sharing and closure; sessions delivered by a board-certified psychodramatist or supervised trainee.	Group; in-person	Two psychodrama sessions per week (approximately 1.75 h + 2.75 h; ~4.5 h/week); mean attendance approximately 4.5 sessions per participant (range 2–9 pre-pandemic; 2–8 during-pandemic).	Significant pre–post reductions were reported in PTSD symptoms (PCL) and depressive symptoms (PHQ-9) in both cohorts. Post-treatment outcomes were comparable across cohorts after controlling for baseline differences. Improvements in depressive symptoms were greater than those observed in a benchmark group of inpatients who did not participate in psychodrama programming (facility comparison).
21	Grigorescu et al. (2020)	Romania	Hospital nurses presenting with medium to high levels of burnout; *N* = 23 (experimental group *n* = 11; no-intervention control group *n* = 12).	Non-randomized controlled pre–post study comparing an intervention group with a no-intervention control group.	Psychodrama (described as a unity-oriented psychotherapeutic approach).	Core (psychotherapeutic sessions used classical psychodrama techniques)	Classical psychodrama techniques were reported; specific enactment methods were not itemized (NR).	Group, in-person	Weekly group sessions lasting approximately 150 min; total number of sessions NR.	Post-intervention reductions were reported in burnout (personal burnout and burnout related to working conditions), along with decreases in depressive and anxiety symptoms in the experimental group compared with the control group.
22	Jaaniste et al. (2015)	Australia	Community-dwelling older adults with mild to moderate dementia (predominantly Alzheimer’s disease); *N* = 13 completers (age range 61–88 years). Quantitative sample: drama therapy group *n* = 4; comparison “normal activity” (movie-watching) group *n* = 9. Two additional drama therapy participants contributed qualitative data only.	Pilot mixed-methods, non-randomized comparative study; drama therapy compared with a non-therapeutic activity control; no random assignment.	Drama therapy	Core (drama-based enactment constituted the tested intervention).	Structured drama therapy sessions including warm-up games/exercises, pair work, group improvisation, reflection, de-roling, and closure; methods such as Developmental Transformations (DvT), story making, role playing, improvisation of personal stories; use of reminiscence objects and photographs.	Group, in-person	Sixteen group sessions, 90 min each, delivered weekly across two 8-week blocks with a 3-week break between blocks.	Quality of life (QoL-AD) scores increased in the drama therapy group and decreased in the comparison group; however, the between-group difference in change scores was not statistically significant. Qualitative findings indicated enhanced emotional expression, communication, and engagement with well-being–related themes among drama therapy participants.
23	Gulassa et al. (2019)	Brazil	Adults with primary DSM-5 excoriation (skin-picking) disorder seeking outpatient treatment; convenience sample *N* = 38 recruited, *N* = 19 treated (psychodrama group therapy *n* = 10; support group therapy *n* = 9).	Open-label pilot comparative study with non-randomized group allocation; two parallel group interventions (psychodrama vs. support therapy); pre–post assessments; clinicians not blinded.	Psychodrama group therapy (PGT) compared with support group therapy (SGT).	Core in (psychodramatic enactment constituted the primary therapeutic mechanism in the experimental condition); comparator involved non-enactment supportive therapy	Psychodramatic exercises involving dramatic exploration of significant relationships; structured session content alternating focus on interpersonal interaction patterns and emotional processing; session-by-session program described.	Group, in-person (outpatient setting)	Planned 20-session group program; mean treatment compliance approximately 13 sessions completed in both groups.	Both groups showed improvements over time in excoriation severity (self-report and clinician ratings) and social adjustment, with no significant time × group interaction (no between-group differences). No significant changes were observed in anxiety, depression, or emotion regulation. Baseline emotion dysregulation was associated with excoriation severity and psychosocial functioning at baseline and post-treatment.
24	Dehnavi et al. (2016)	Iran	Female adults with relapsing–remitting multiple sclerosis (RRMS) in the suppression phase, with independent mobility; age range 20–52 years; additional eligibility criteria reported; *N* = 20 (experimental group *n* = 10; no-intervention control group *n* = 10).	Quasi-experimental controlled pre–post study with 2-month follow-up; convenience sampling; non-randomized group allocation despite use of the term “randomly divided.”	Group psychodrama based on classical psychodrama principles.	Core (psychodrama constituted the primary therapeutic intervention).	Role-play–based psychodrama techniques explicitly described, including doubling (duplication), role reversal, mirroring, future projection, monologue, self-actualization, and related behavioral exercises.	Group, in-person	Twelve group sessions, 2 h each, delivered over 6 weeks.	Depressive symptoms assessed with the Beck Depression Inventory (BDI) showed a marked reduction in the psychodrama group from pre-test to post-test, maintained at follow-up. ANCOVA indicated a significant between-group effect favoring psychodrama.
25	Gonzalez et al. (2018)	Portugal	Adult psychotherapy clients attending a university clinic psychodrama group; *N* = 5 (3 women, 2 men; age range 27–48 years). All participants held university degrees and were professionally active at treatment entry.	Longitudinal mixed-methods study using the Hermeneutic Single Case Efficacy Design (HSCED); repeated quantitative assessments across four time points (approximately every 6 months) combined with qualitative process analyses. One participant (“John”) was selected for an adjudicated single-case analysis due to the completeness of available data; no control group.	Psychodrama group psychotherapy.	Core (psychodrama constituted the psychotherapy model and primary treatment process).	Psychodramatic enactments described through session events and named techniques, including role reversal, Magic Shop, social atom, sculpture, dramatizations, and group games.	Group, in-person; typical group size 4–7 participants; two therapists	Weekly group sessions of approximately 2 h. Total treatment length varied by participant; the group-level dataset spanned approximately 2 years, while the adjudicated single case involved 5 years of therapy with a 6-month follow-up.	At the group level, trends of improvement were observed over time, including reduced psychological distress (CORE-OM), decreased problem-related distress (Personal Questionnaire), and increased spontaneity (SAI-R). In the adjudicated single case, changes met criteria for reliable and clinically significant improvement across outcomes and were supported by qualitative process data (Helpful Aspects of Therapy; Client Change Interview), including maintenance at 6-month follow-up.
26	Sevi et al. (2020)	Turkey	Inpatients with chronic schizophrenia receiving care in a chronic inpatient psychiatric service; *N* = 31 (12 women, 19 men). Participants were allocated to two gender-separated psychodrama groups.	Uncontrolled pre–post study with no control group (acknowledged as a limitation by the authors); pre- and post-intervention assessments; no follow-up.	Psychodrama group psychotherapy delivered alongside stable standard antipsychotic treatment.	Core (psychodrama sessions constituted the primary psychotherapeutic component; no comparator condition).	Warm-up activities and games to establish safety and facilitate enactment; protagonist-centered scenes; role-based “as-if” group activities (e.g., group tree/roles, wax sculpture, group forest); termination-focused enactments. Classical psychodramatic techniques (e.g., role reversal, doubling) were not systematically itemized (NR).	Group, in-person (inpatient setting); two gender-separated groups led by a group leader and assistant leader	Nine weekly group sessions; session duration NR. Sociometric assessments were conducted during sessions 5 and 17.	Significant pre–post improvements were reported in psychotic symptoms and overall quality of life. Outcome patterns differed by gender: the female group showed significant reductions in general psychopathology and increased quality of life, whereas the male group showed significant reductions in positive and negative symptoms and PANSS total scores, alongside improved quality of life. Depressive symptoms (Calgary Depression Scale) decreased slightly but did not reach statistical significance. Sociometric indices (e.g., sociometric status, group harmony) improved, with increased elections and reduced rejections and isolates. No follow-up assessment was conducted

Core indicates that role playing or enactment constituted the primary and recurring psychotherapeutic mechanism of the intervention. Integrated indicates that role playing was embedded as a recurring component within a broader psychotherapeutic model (e.g., CBT, EFT), but did not constitute the sole treatment framework.

NR indicates that the information was not reported or not explicitly described in the original study.

Study designs are reported as described by the authors; results should be interpreted in light of the corresponding methodological rigor and risk-of-bias assessments reported in Table 2.

BDI, Beck Depression Inventory; CORE-OM, Clinical Outcomes in Routine Evaluation–Outcome Measure; HAMD, Hamilton Depression Rating Scale; LSAS, Liebowitz Social Anxiety Scale; PCL, PTSD Checklist; QoL, quality of life; SAI-R, Spontaneity Assessment Inventory–Revised; TAS-20, Toronto Alexithymia Scale; QLS, Quality of Life Scale.

**Table 2:** Risk of bias and methodological quality of included studies.

Randomized controlled trials (RoB 2)							
Study	Randomization	Deviations	Missing outcome data	Measurement	Selective reporting	Overall	Notes
Elkarif et al. (2025)	High risk	Some concerns	Some concerns	Some concerns	Some concerns	High risk	Randomization and allocation concealment unclear; reliance on self-report outcome measures.
Yu et al. (2022)	Some concerns	Some concerns	High risk	Some concerns	Some concerns	High risk	Imbalanced attrition across groups and incomplete reporting of allocation procedures and analytic strategy.
Bardideh et al. (2025)	Some concerns	Some concerns	Some concerns	Some concerns	Some concerns	Some concerns	Randomization stated; limited information on allocation concealment and blinding; outcomes primarily self-reported; follow-up assessment reported.
Gulassa et al. (2025)	Low risk	Some concerns	Some concerns	Some concerns	Low risk	Some concerns	Block randomization using an external random number generator; active control condition; psychotherapy not blinded; intention-to-treat analysis stated but handling of missing data not fully specified; majority of outcomes self-reported; trial registered

Non-randomized comparative/intervention studies (ROBINS-I)									
Study	Confounding	Selection	Classification	Deviations	Missing data	Measurement	Reporting	Overall	Notes
Dehnavi et al. (2016)	Serious	Serious	Moderate	Moderate	Low	Moderate	Moderate	Serious	Convenience sample; random division reported but concealment and confounding control limited.
Keisari and Palgi (2016)	Serious	Serious	Moderate	Moderate	Moderate	Moderate	Moderate	Serious	Non-random allocation; baseline differences and confounding likely.
Terzioğlu and Özkan (2018)	Serious	Serious	Moderate	Serious	Moderate	Moderate	Moderate	Serious	Non-random allocation; baseline differences and confounding likely.
Grigorescu et al. (2020)	Serious	Serious	Low	Moderate	Moderate	Moderate	Moderate	Serious	Volunteer sample; non-random assignment; limited adjustment for confounding.
Jaaniste et al. (2015)	Serious	Serious	Moderate	Serious	Moderate	Moderate	Moderate	Serious	Non-equivalent comparison groups; very small n; site confounding.
Gulassa et al. (2019)	Serious	Serious	Moderate	Moderate	Moderate	Moderate	Moderate	Serious	Open-label pilot; non-randomized comparative design; limited blinding.

Uncontrolled pre–post studies (NIH Before–After tool).		
Study	Overall quality	Notes
Biolcati et al. (2017)	Fair	One-group pre–post; no control.
Biolcati et al. (2023)	Fair	Tele-based; self-report outcomes.
Şener (2022)	Poor	Single-group; small sample; reporting limitations.
Gamoneda et al. (2025)	Poor	Open trial; no comparator; limited reporting for some outcomes.
Erbay et al. (2018)	Poor	No control; small sample; limited protections against bias.
Abeditehrani et al. (2020)	Poor	Very small uncontrolled pilot (*n* = 5).
Sevi et al. (2020)	Fair	Inpatient pre–post only; no control; outcomes partly objective but no blinding.
Doomen (2018)	Fair	Exploratory single-group study; incomplete reporting of dose and procedures; no control.

Qualitative studies (CASP)		
Study	Overall quality	Notes
Menichetti et al. (2016)	Moderate	Small sample; reflexivity variably reported.
Biancalani et al. (2021)	Moderate	Convenience sample; limited reflexivity detail.

Mixed-methods studies (MMAT 2018)		
Study	Overall quality	Notes
Testoni et al. (2018)	Moderate	Mixed-methods idiographic design; no control.
Testoni et al. (2020)	Moderate	Exploratory; small sample; uncontrolled quantitative component.
Giacomucci et al. (2025)	Moderate	Naturalistic inpatient study; uncontrolled quantitative component.

Case reports/case series and text/opinion papers (JBI)		
Study	Overall quality	Notes
Keisari et al. (2023)	Moderate	Case-based report; limited control and small n.
Gonzalez et al. (2018)	Moderate	HSCED with small N.

Risk of bias and methodological quality were assessed at the study level using design-appropriate, validated appraisal tools. All assessments were conducted on full-text articles. Overall judgments reflect the highest-risk domain materially affecting internal validity and are reported alongside domain-level ratings in this table.

RoB 2, Cochrane risk of bias 2 tool for randomized trials (low risk, some concerns, high risk). ROBINS-I, risk of bias in non-randomized studies of interventions (low, moderate, serious, critical). NIH before–after, NIH/NHLBI quality assessment tool for before–after studies with no control group (good/fair/poor). CASP, critical appraisal skills programme qualitative checklist. MMAT (2018), mixed methods appraisal tool. JBI, Joanna Briggs Institute critical appraisal tools. Overall judgments follow each tool’s guidance and reflect the highest-risk domain materially affecting internal validity rather than a numerical aggregation of domains.

### 2.5 Risk of bias and quality assessment

Methodological quality and risk of bias of the included studies were formally assessed using design-appropriate tools. Randomized controlled trials were evaluated with the Cochrane Risk of Bias 2 (RoB 2) tool (Sterne et al., 2019). Non-randomized comparative studies were assessed using the Risk of Bias In Non-randomized Studies of Interventions (ROBINS-I) tool (Sterne et al., 2016). Uncontrolled pre–post studies, case series, and qualitative studies were appraised using appropriate critical appraisal frameworks adapted to their respective designs. Two reviewers independently conducted the risk-of-bias assessments for all included studies. Discrepancies were resolved through discussion, and when consensus could not be reached, a third reviewer was consulted. For each study, an overall risk-of-bias judgment was derived based on the domain-level ratings provided by the respective tools. Overall judgments followed each tool’s guidance and reflect the domain judged to pose the greatest threat to internal validity, rather than an average or numerical aggregation of domains. The results of the quality assessment are summarized in the main text and reported in detail in a dedicated table (Table 2), which presents both overall judgments and domain-specific ratings for each included study.

## 3 Results

### 3.1 Clinical outcomes

In light of the predominance of non-randomized and uncontrolled designs, outcome patterns reported in the following sections should be interpreted as preliminary trends rather than estimates of treatment efficacy. Observed outcome patterns varied systematically by study design and methodological rigor, with more circumscribed and condition-specific effects reported in higher-rigor studies. Across the included studies, role-playing interventions were associated with improvements in multiple clinical domains. A first and most consistently reported outcome concerned the reduction of psychological symptoms across heterogeneous clinical conditions. For example, in interventions targeting depression or stress-related difficulties, the enactment of personal problems or interpersonal roles appeared to provide patients with an emotionally safe context to express and reorganize critical experiences, leading to significant symptom decreases (Erbay et al., 2018; Dehnavi et al., 2016). In Yu et al. (2022), the psychodramatic process facilitated the externalization of perceived psychological difficulties, which was associated with reductions in depressive symptoms and corresponded to measurable changes in functional neural connectivity, suggesting that embodied enactment may support neuropsychological processes underlying recovery. Notably, even in randomized trials, effects were often domain-specific and accompanied by methodological limitations, including incomplete reporting of allocation procedures and reliance on self-report outcomes. A second cluster of outcomes involved improvements in emotional expression and regulation. These changes were especially prominent in interventions focusing on grief or chronic pain. In Şener (2022), for instance, women with fibromyalgia engaged in dramatization of emotionally salient roles, which enabled them to articulate previously unexpressed affect and reorganize their emotional responses. Similarly, in grief-focused empty-chair work (Gamoneda et al., 2025), the structured enactment of a dialogical exchange with the absent other promoted emotional activation and subsequent integration, resulting in decreased grief-related distress. In both cases, role enactment functioned as an experiential scaffold: it made emotions more accessible and symbolically workable, thereby fostering regulatory capacity. Interpersonal and social functioning also improved across several studies, particularly in psychodramatic group contexts. These effects appear tied to relational mechanisms intrinsic to role-playing–such as role reversal, doubling, and mirroring–which allow participants to embody the perspectives of others and receive immediate feedback from the group. Keisari and Palgi (2016) similarly showed that role-based life-review processes in older adults strengthened social connectedness and interpersonal flexibility. Tele-based adaptations demonstrated that these relational processes can occur even in digital formats, as shown by Elkarif et al. (2025), where remote dramatization still elicited empathy and cohesion among participants with limited mobility. Another set of studies highlighted how role-playing fosters empowerment, self-awareness, and meaning-making. In Testoni et al. (2018), meaning-centered psychodrama enabled participants to enact existential themes and explore symbolic representations of loss, producing increased acceptance and psychological integration. Similarly, cancer patients in Menichetti et al. (2016) reported heightened agency and self-understanding after embodying personally significant roles, suggesting that role enactment enhances reflective capacity and supports identity reorganization. These outcomes may stem from the opportunity to embody alternative versions of oneself, test new narratives, and observe oneself from a distanced perspective. In a smaller subset of studies, role-playing produced biopsychological effects. The reduction of salivary cortisol observed by Erbay et al. (2018) suggests that embodied expression through dramatization may attenuate physiological stress responses; likewise, the neurofunctional changes reported by Yu et al. (2022) indicate that action-based emotional processing might engage neural circuits involved in self-referential thinking and emotional regulation. These findings remain preliminary but point toward potential psychophysiological pathways through which role enactment may promote therapeutic change. When outcomes were examined in relation to theoretical orientations, psychodramatic interventions showed improvements, spanning emotional, interpersonal, and symptomatic domains. Integrative approaches-such as the combination of psychodrama with Cognitive Behavioral Therapy (CBT), (Abeditehrani et al., 2020) or Emotion-Focused Therapy, EFT, (Gamoneda et al., 2025)–tended to generate more targeted effects, particularly in emotion regulation, empathic accuracy, and fear of negative evaluation. Other studies, including those using behavioral rehearsal in CBT formats, produced focused changes in specific behavioral or interpersonal competencies. Taken together, the review indicates that symptom reduction and improvements in emotional regulation were the most consistently observed outcomes across both group and individual interventions. Although comparisons across orientations should be interpreted cautiously due to methodological heterogeneity, the pattern suggests that the therapeutic impact of role-playing may depend not only on the clinical population but also on the specific enactment procedures and theoretical assumptions shaping the intervention. Importantly, not all studies reported statistically significant or differential effects. In several investigations, improvements were observed across time but did not differ between intervention and comparison conditions (e.g., Jaaniste et al., 2015; Gulassa et al., 2019), while other outcomes showed non-significant or modest changes, particularly for depressive symptoms or global quality of life (e.g., Sevi et al., 2020; Abeditehrani et al., 2020). These null or mixed findings further underscore the preliminary nature of the available evidence.

### 3.2 Type, clinical population, and intervention

A total of 26 studies published between 2015 and 2025 met the inclusion criteria. Study designs were heterogeneous and predominantly non-randomized. Approximately four studies employed randomized controlled designs, six used quasi-experimental or non-randomized comparative designs, while the majority relied on uncontrolled pre–post, mixed-methods, qualitative, or case-based approaches. Sample sizes ranged from single-case reports to naturalistic studies with over 120 participants. Counts should be interpreted as approximate due to heterogeneity in reporting and hybrid study designs. The included studies involved a diverse range of adult clinical populations, including individuals with depression (Erbay et al., 2018; Yu et al., 2022), substance use disorders (Testoni et al., 2020; Giacomucci et al., 2022, 2025), oncological conditions (Menichetti et al., 2016), infertility (Terzioğlu and Özkan, 2018), fibromyalgia (Şener, 2022), and complicated grief (Gamoneda et al., 2025). Additional studies focused on older adults (Keisari and Palgi, 2016; Elkarif et al., 2025; Jaaniste et al., 2015), university students (Biolcati et al., 2017, 2023), health professionals (Grigorescu et al., 2020), and populations presenting with eating-related, trauma-related, or body-focused repetitive behaviors (Gulassa et al., 2019; Giacomucci et al., 2025). Most studies employed group-based interventions (approximately three quarters of the sample), while individual formats were used in a smaller subset of studies. Individual role-playing interventions—predominantly involving empty-chair or dialogical enactment techniques—were most commonly applied to grief-related difficulties, trauma-related experiences, or interpersonal conflicts. Intervention duration varied substantially across studies. One study examined a single-session intervention, while others implemented brief interventions ranging from 3 to 12 sessions, medium-term interventions of 13–30 sessions, or long-term interventions exceeding 30 sessions or extending beyond 6 months. These categories should be interpreted as approximate, as reporting of session number and intervention length varied considerably across studies. Weekly sessions were the most common delivery schedule. Session duration typically ranged from approximately 75 to 120 min for group interventions and from 50 to 90 min for individual formats. Across studies, commonly reported enactment techniques included role reversal, doubling, mirroring, symbolic enactment, and dramatization of emotionally salient material. Several psychodrama studies explicitly described warm-up, action, and sharing phases consistent with classical Morenian practice; however, the level of procedural detail and specificity regarding role-playing methods varied substantially across the included studies. Procedural details were extracted and coded only when explicitly reported by the study authors; the absence of detail reflects reporting limitations rather than absence of enactment.

### 3.3 Theoretical framework

Across the 26 included studies, psychodrama-based interventions represented the predominant theoretical framework (approximately 77% of studies), typically grounded in Morenian principles such as warm-up, action, and sharing. A smaller number of studies employed drama therapy models, integrative approaches combining psychodramatic enactment with CBT or EFT principles, or chair-based experiential techniques. Given the limited number of studies outside psychodramatic traditions, conclusions regarding the relative effectiveness of different theoretical orientations remain tentative. Three studies integrated psychodramatic enactments with cognitive-behavioral components, embedding behavioral rehearsal or cognitive restructuring within action-based procedures. One study reflected emotion-focused principles, using role enactment to facilitate emotional activation and processing. Two studies adopted humanistic or person-centered orientations, particularly in meaning-centered or expressive drama therapy interventions. These findings indicate that current research overwhelmingly conceptualizes role-playing within psychodramatic or experiential frameworks, while applications in other orientations remain scarce. When theoretical framework and format were considered together, psychodramatic group interventions were predominantly used for complex, multi-determined conditions such as substance use disorders, chronic medical illness, and late-life adjustment, whereas individual role-playing formats (mainly empty-chair techniques) were more frequently applied to grief, trauma-related difficulties, and interpersonal conflicts. This pattern could suggest that group-based role-playing is often chosen when relational and sociometric dimensions are central, while individual enactments tend to be used for focused emotional processing and intrapersonal themes.

### 3.4 Emerging trends and gaps

One emerging trend concerns the expansion of digital formats. Experiences gained during and after the pandemic suggest that key therapeutic processes may be preserved in online or hybrid environments, including the sharing of emotional experiences, the maintenance of interpersonal relationships, and a sense of therapeutic presence. Biolcati et al. (2023) and Elkarif et al. (2025) highlight that the transition to digital does not necessarily compromise the typical change processes of in-person work, suggesting significant potential for expanding access to populations with mobility difficulties or geographical barriers. In parallel, there is growing interest in integrative models that combine psychodramatic actions with different theoretical frameworks, including CBT, EFT, and narrative approaches. These studies (e.g., Abeditehrani et al., 2020; Gamoneda et al., 2025, and Keisari et al., 2023) show how enactment can be used to enhance specific change processes already envisaged by reference models, such as gradual exposure, cognitive restructuring, or transformation of maladaptive primary emotions. Another emerging trend involves the application of role-playing to clinical groups traditionally less represented in psychotherapeutic literature. Recent studies show increasing attention to populations with chronic diseases, forensic contexts, and elderly people or those with cognitive impairments, indicating that enactment can function as a tool not confined to specific domains, but can be modulated to respond to very different needs, enhancing both symbolic and interpersonal dimensions of the therapeutic experience. Alongside these developments, the review also highlights four possible gaps that deserve attention to guide future research. Firstly, almost all available evidence comes from psychodramatic frameworks, while applications based on other models, based on the criteria employed, remain little cited. Secondly, the strong methodological heterogeneity–which includes significant differences in samples, outcome measures, definition of role-playing procedures, and use of control groups–prevents drawing solid conclusions on the relative effectiveness of interventions. Many studies present small samples and designs without randomization, making it necessary to interpret the results as preliminary indicators rather than definitive evidence. A third gap emerging from the review concerns the scarcity of systematic follow-ups and the almost total absence of information on the therapists involved, including training, technical competence, and adherence to protocols. Finally, despite several theoretical frameworks suggesting the usefulness of experiential tasks between sessions, none of the studies examined and included in the review mentioned the use of role assignments outside the session.

### 3.5 Findings stratified by study design and methodological rigor

Given the substantial heterogeneity of study designs, findings were examined according to methodological rigor, as follows:

#### 3.5.1 Randomized controlled trials (RCTs)

Four studies employed randomized controlled designs to evaluate psychodramatic or enactment-based interventions, focusing on outcomes such as symptom severity, emotional regulation, and interpersonal functioning. Across these trials, improvements were reported in at least one outcome domain relative to baseline or comparison conditions. However, effect sizes were not consistently reported, and follow-up was inconsistent (present in some RCTs, absent in others).

#### 3.5.2 Quasi-experimental and non-randomized comparative studies

Studies adopting quasi-experimental or non-randomized comparative designs commonly reported improvements in symptom reduction, interpersonal functioning, and emotional processing following role-play–based interventions. Nonetheless, the absence of randomization and the presence of baseline group differences limit the interpretability of between-group effects.

#### 3.5.3 Uncontrolled pre–post and case-based studies

Several studies relied on uncontrolled pre–post designs, case series, or qualitative methodologies. These investigations consistently described changes in emotional expression, self-awareness, and relational functioning. While informative regarding feasibility and potential mechanisms of change, such designs do not allow estimation of treatment effects and should therefore be considered exploratory. Overall, higher-rigor designs tended to report more circumscribed and outcome-specific effects, whereas lower-rigor studies primarily contributed descriptive and process-oriented findings.

### 3.6 Risk of bias and methodological quality

As shown in Table 2, the methodological quality of the included studies was heterogeneous and generally limited. None of the randomized controlled trials were rated at low risk of bias; instead, all presented either some concerns or high risk, primarily due to limitations in the randomization process, incomplete handling of missing outcome data, and reliance on self-reported measures. Importantly, the use of a randomized design did not automatically translate into low risk of bias, as all identified RCTs exhibited at least one RoB 2 domain rated as some concerns or high risk. Most non-randomized comparative studies were judged to be at serious risk of bias according to the ROBINS-I tool, mainly due to confounding and selection bias related to non-random allocation procedures. Uncontrolled pre–post studies and case-based designs were predominantly rated as fair to poor quality, reflecting the absence of control groups and limited protection against threats to internal validity. Qualitative and mixed-methods studies generally demonstrated moderate methodological quality and contributed primarily process-oriented and experiential evidence.

## 4 Discussion

This systematic review synthesized a decade of empirical research on role-playing interventions in adult psychotherapy, highlighting an intervention modality applied across diverse clinical populations, formats, and theoretical frameworks. Across studies, role-playing interventions were reported to be associated with improvements in emotional, interpersonal, and symptomatic domains, suggesting that enactment-based methods may support therapeutic change through experiential and relational processes. A central contribution of this review lies in the identification of recurrent mechanisms of change across heterogeneous designs. Role-playing appears to facilitate emotional activation, perspective-taking, and reflective integration by allowing patients to externalize internal experiences, embody alternative roles, and observe themselves from a distanced position. These processes were evident across group and individual formats, as well as in both in-person and remote interventions, underscoring the flexibility of enactment-based methods in addressing a wide range of clinical presentations. At the same time, the interpretation of these findings is constrained by methodological limitations. Most studies employed non-randomized, uncontrolled, or exploratory designs, and even higher-rigor studies were characterized by small samples, incomplete reporting of allocation procedures, and infrequent follow-up assessments. More rigorous designs tended to report more circumscribed and outcome-specific effects, whereas qualitative and case-based studies primarily emphasized experiential, relational, and meaning-making outcomes. This pattern suggests that study design influences not only the strength of causal inference but also the type of outcomes that are most readily captured. Although therapist-related variables were not systematically extracted or analyzed in the present review, their potential relevance warrants consideration. While some studies reported descriptive information on therapist training or professional background, therapist-related variables were not reported in a systematic or standardized manner, precluding any formal synthesis of therapist effects. As emphasized in the broader psychotherapy literature, treatment outcomes are not solely attributable to specific techniques but are substantially shaped by therapist characteristics, relational skills, and clinical competence (Miller and Moyers, 2021). This consideration is particularly salient for role-playing interventions, which involve complex enactment procedures, emotional activation, and identity experimentation. The effectiveness of role-playing likely depends on the therapist’s ability to structure enactments, maintain a safe therapeutic frame, regulate emotional arousal, and facilitate reflective integration following action-based work. Insufficient training or limited adherence to role-playing procedures may result in substantial variability in therapeutic impact, even when similar techniques are employed. Accordingly, future research on enactment-based interventions should systematically assess and report therapist-related variables, including training background, experience with role-playing methods, and adherence to intervention protocols, in order to better disentangle technique-specific effects from clinician-related influences. Although we summarized findings by study design and reported risk of bias, studies were not excluded based on methodological quality (Iudici et al., 2015; Iudici et al., 2024). Accordingly, conclusions should reflect the differential evidential weight associated with different designs, and reported improvements should be interpreted as indicating promising trends rather than definitive evidence of efficacy. Therefore, the present review should be understood as a systematic synthesis of outcome patterns and methodological trends, rather than as a conclusive effectiveness review in the strict meta-analytic sense. In addition, we adopted a broad definition of role-playing in order to synthesize findings across multiple theoretical approaches. It is possible that the relevant literature is more extensive than captured by the present criteria, as studies employing enactment or role-assignment procedures may not explicitly label these techniques as role-playing. This definitional choice should be considered when interpreting the scope of the present review. Overall, the findings suggest that role-playing represents a promising and versatile therapeutic modality. However, the current evidence base remains methodologically heterogeneous and insufficient to support strong causal claims. Future research should prioritize adequately powered randomized controlled trials, clearer reporting of allocation and intervention procedures, systematic follow-up assessments, and greater attention to therapist-related variables. Such efforts will clarify the conditions under which role-playing interventions can be effectively and safely integrated into evidence-based psychotherapeutic practice.

## 5 Conclusion

This systematic review mapped a decade of empirical research on role-playing interventions in adult psychotherapy, providing an overview of how enactment-based methods have been applied across diverse clinical populations, settings, and theoretical frameworks. Overall, the available literature suggests that role-playing has been investigated as a flexible and experiential therapeutic modality with potential relevance for emotional, interpersonal, and behavioral change. At the same time, the strength and generalizability of these findings are limited by the marked heterogeneity of the evidence base. Although a small number of randomized and comparative studies were identified, much of the literature consists of small-sample, non-randomized, or exploratory designs. Accordingly, reported improvements should be interpreted as promising trends rather than definitive evidence of efficacy, and conclusions must be weighted according to study design and methodological rigor. Importantly, therapist-related variables—including training, adherence to the intervention model, and competence in managing emotionally activating procedures—were rarely reported, despite their likely influence on outcomes. This gap limits the interpretability and replicability of findings and highlights the need to consider therapist effects when evaluating role-playing interventions. Future research should therefore move beyond predominantly exploratory applications by adopting methodologically robust designs, including adequately powered randomized controlled trials across a wider range of theoretical orientations. Greater attention to the standardized description of enactment procedures, systematic follow-up assessments, and the reporting of therapist-related variables will be essential to clarify the conditions under which role-playing interventions can be integrated with stronger evidential support into evidence-based psychotherapeutic practice.

## Data Availability

The raw data supporting the conclusions of this article will be made available by the authors, without undue reservation.
